# Microbiological diagnosis of pulmonary invasive aspergillosis in critically ill patients with severe SARS-CoV-2 pneumonia: a bronchoalveolar study

**DOI:** 10.1186/s12941-023-00626-7

**Published:** 2023-10-10

**Authors:** Ángel Estella, Ignacio Martín-Loeches, María Recuerda Núñez, Clara González García, Liliana Marcela Pesaresi, Alvaro Antón Escors, Maria Dolores López Prieto, Juan Manuel Sánchez Calvo

**Affiliations:** 1 Intensive Care Unit University Hospital of Jerez, University of Cádiz. INIBiCA, Jerez de la Frontera, Spain; 2 Department of Intensive Care Medicine, Multidisciplinary Intensive Care Research Organization (MICRO), St James’ Hospital, Dublin, Ireland; 3 Intensive Care Unit University Hospital of Jerez, INIBiCA, Jerez de la Frontera, Spain; 4 Medical School University of Cádiz, Cadiz, Spain; 5 Infectious diseases and Microbiology, Unit Hospital Universitario de Jerez, INIBiCA, Jerez de la Frontera, Spain

**Keywords:** CAPA, covid-19, Galactomannan, Bronchoalveolar lavage, Lateral flow, Fungal culture, Sensitivity, Specificity

## Abstract

**Background:**

Diagnosing COVID-19-associated pulmonary aspergillosis (CAPA) can be challenging since radiological and clinical criteria in the critically ill patient are nonspecific. Microbiological diagnostic support is therefore crucial. The aim of this study was to document the incidence of aspergillosis using bronchoalveolar lavage (BAL) as the diagnostic method and to determine the performance of the current mycological diagnostic tests most widely used for the diagnosis of CAPA, together with evaluation of the Asp lateral flow device (LFD).

**Methods:**

Prospective cohort study conducted between March 2020 and June 2022. Inclusion criteria were critically ill patients admitted to the ICU with SARS-CoV-2 pneumonia requiring invasive mechanical ventilation. Diagnostic bronchoscopy and BAL were performed at the beginning of invasive mechanical ventilation. The sensitivity, specificity, positive and negative predictive value (PPV and NPV), positive and negative likelihood ratio (LR + and LR-) of BAL culture, direct examination with calcofluor white stain, ELISA (Platelia) and LFD (AspLFD) for detection of galactomannan (GM) were evaluated. Aspergillus-qPCR was applied when discrepancies between diagnostic tests arose.

**Results:**

Of the 244 critically ill patients with SARS-CoV-2 pneumonia admitted to the ICU, the majority (n = 200, 82%) required invasive mechanical ventilation. Diagnostic bronchoscopic procedures were performed in 160 patients (80%), who were enrolled in this study. The incidence of CAPA was 18.7% (n = 30). LFD-GM demonstrated a sensitivity of 84%, specificity of 99%, PPV 94%, NPV 97%, LR(+) of 84, and LR(-) of 0.16. At GM-ELISA indices of ≥ 0.5 and ≥ 1.0, sensitivity was 92% and 79%, specificity was 95% and 99%, PPV 76% and 91%, NPV 99% and 96%, LR(+) 18 and 79, and LR(-) 0.08 and 0.21, respectively. The optimal cut-off index from the ROC curve was 0.48, with sensitivity of 95% and specificity of 95%.

**Conclusions:**

Using a diagnostic strategy based on bronchoscopy and BAL, we documented a high incidence of pulmonary aspergillosis in patients with severe SARS-CoV-2 pneumonia. Asp-LFD showed moderate sensitivity and excellent specificity, with a high PPV, and could be used for rapid diagnosis of patients with suspected CAPA.

## Background

Recurrent coronavirus disease 2019 (COVID–19) pandemic outbreaks have placed a tremendous burden on healthcare, especially in patients admitted to the ICU requiring invasive mechanical ventilation [1, 2]. It has been suggested that the development of co-infections and/or secondary healthcare-associated infections is one of the contributory causes of poor prognosis in the ICU [3–5]. There is currently some controversy about the benefit of corticosteroid treatment in severe forms of SARS-CoV-2 pneumonia [6], as an increase in fungal infections has been reported with this treatment. COVID-19-associated pulmonary aspergillosis (CAPA) has been reported in previous studies, with varying mortality rates [7–12]. It is likely that the different reported rates are mainly due to the different definitions of CAPA and diagnostic methods. It is generally recognized that diagnosing CAPA is not straightforward and different classifications have been proposed to make a determination as “proven”, “probable”, or “possible”. Based on previously published reports, the use of bronchoscopy in invasively mechanically ventilated patients is not widespread. Unfortunately, in many cases of CAPA reported in the last two years, not even one invasive respiratory specimen was obtained [13, 14].

We hypothesize that invasive sampling is associated with increased diagnostic accuracy. We analyzed the diagnostic value of most of the latest microbiological techniques for the diagnosis of CAPA using invasive diagnostic methods. The aim of this study is to document the incidence of aspergillosis using bronchoalveolar lavage (BAL) as the diagnostic method, and to determine the performance of the mycological diagnostic tests most widely used for diagnosis of CAPA, together with evaluation of the Asp lateral flow device (LFD).

## Materials and methods

### Study population

This was a prospective observational study conducted in a major teaching hospital. Critically ill patients with SARS-CoV-2 pneumonia admitted to the ICU since the onset of the COVID-19 pandemic were included. The study period ran from March 2020 to June 2022. Patients were eligible if they were: (1) 18 years or older; (2) receiving invasive ventilation for COVID–19 SARS–CoV-2 infection confirmed by RT–PCR. Exclusion criteria were patients not requiring invasive mechanical ventilation, or in whom bronchoscopy was deemed unsafe. Diagnostic fibrobronchoscopy was performed in all mechanically ventilated patients on admission to the ICU or with suspected secondary infection. No patients received antifungal therapy prior first bronchoscopy bronchoalveolar lavage.

### Samples processed

Bronchoscopy and bronchoalveolar lavage (BAL) was performed with 150 ml of physiological saline solution, divided into three aliquots. The first 20 ml of the BAL was discarded, and a sample of the remaining fluid was collected for microbiological analysis. Fibrobronchoscopy was performed with disposable AMBU aScope™ 4 Broncho Regular OD 5.8 / 2.8 mm bronchoscopes (Copenhagen, Denmark). The bronchoscope was inserted using a special adapter valve to avoid generating aerosols during the procedure. A specialist pulmonologist with 30 years of experience not related to the research study randomly analyzed the BAL samples to perform a quality control; those with few alveolar macrophages and/or excessive numbers of airway-derived cells were considered unsuitable due to the low quality of the sample. No local anesthetics were administered, and aspiration was not attempted through the bronchoscope channel until it was in position at the site to be studied. BAL samples were sent to microbiology the service to be processed.

### Microbiological studies

Qualitative galactomannan (GM) detection, culture on fungal media and calcofluor white staining were performed within the first hours, all tests were performed from the same BAL fluid. All BAL fluids were stored at -80ºC for quantitative GM detection and quantitative PCR (qPCR) assay for detection of the *Aspergillus* genus.

#### Qualitative GM detection

The Aspergillus-specific lateral flow device (AspLFD, OLM Diagnostics, Newcastle upon Tyne, United Kingdom) is a rapid immunochromatography test for the qualitative detection of *Aspergillus* diagnostic GM antigen. The results were reported as weak positive or strong positive in less than one hour.

BAL samples were processed according to the manufacturer’s recommendations [15]. Nevertheless, all BAL samples, both blood-free and bloody, were pretreated.

#### Calcofluor white staining

Direct examination of BAL fluids by fluorescence microscopy used calcofluor white stain (Becton Dickinson) for microscopic observation of filamentous fungi. Staining was positive when we observed septate hyaline hyphae branching at 45 degrees, suggesting *Aspergillus* species. Smear microscopy was performed using potassium hydroxide (KOH) and calcofluor white.

#### Culture on fungal media

BAL fluid was cultured on Sabouraud dextrose agar (SDA) with chloramphenicol (Becton Dickinson) prior to sample processing. For this, 10–20 ml of BAL was centrifuged for 10 min at 3000 rpm. 100 µl of sediment was inoculated onto fungal media. Fungal cultures were incubated at 30ºC for seven days. *Aspergillus* species were identified by matrix-assisted laser desorption/ionization time-of-flight (MALDI-TOF) mass spectrometry and/or using colony characteristics and conidiophore and conidia morphology.

#### Quantitative GM detection

The Platelia *Aspergillus* galactomannan assay (Platelia, Bio Rad) is an enzyme immunoassay (EIA) that detects the GM antigen produced by *Aspergillus* species during active growth. This GM-EIA was performed twice a week on BAL fluid from these patients. Two different cut-off points were considered to determine positive results: GM index ≥ 0.5 ng/ml and ≥ 1.0 ng/ml.

#### Aspergillus-qPCR

In cases where there were discrepancies because only one of the previously described tests was positive, a qPCR assay (*Aspergillus* species ELITe MGB® Kit) was performed for the detection and quantification of the DNA of the *Aspergillus* genus: *Aspergillus niger*, *Aspergillus nidulans*, *Aspergillus terreus*, *Aspergillus flavus*, *Aspergillus versicolor* and *Aspergillus glaucus*. Only copy numbers > 150 of the genus *Aspergillus* (Ct < 36) were considered positive.

### Definitions

#### CAPA

The 2020 ECMM/ISHAM consensus criteria proposed three different grades: possible, probable, and proven [16]. Lung biopsies were not performed in this patient population, so there were no cases of proven CAPA in our cohort. All samples analyzed involved BAL fluid, as classifying patients as possible CAPA was not appropriate. Probable CAPA, according to the consensus criteria, depends on a single piece of mycological evidence. In this study, probable CAPA was defined on the basis of at least two positive diagnostic tests:

1) Detection of GM by AspLFD and/or EIA and BAL culture-positive for *Aspergillus* species or with calcofluor white stain suggestive of *Aspergillus* species.

2) Detection of GM by AspLFD and/or EIA and/or BAL culture-positive for *Aspergillus* species and positive *Aspergillus*-qPCR;

#### Negative case

Patients were defined as negative for CAPA when all the diagnostic tests returned negative results or only one test was positive.

### Statistical analysis

For the baseline characteristics of the study population, data were reported as absolute counts (%) or as medians [25th-75th percentile] and p values were assessed by Mann Whitney U test, Pearson’s χ^2^ test or Fisher’s exact test.

The sensitivity, specificity, positive predictive value (PPV), negative predictive value (NPV), positive likelihood ratio (LR(+)) and negative likelihood ratio (LR(-)) of AspLFD, Platelia *Aspergillus* galactomannan assay, culture on SDA with chloramphenicol and calcofluor white staining were analyzed.

In a receiver operating characteristic (ROC) curve analysis for the Platelia *Aspergillus* galactomannan assay, the area under the curve (AUC), including 95% confidence intervals (CI), was examined to assess the ability of the diagnostic test to discriminate between patients with CAPA and critically ill patients without CAPA. The Youden index was applied to determine the optimal ROC curve cut-off value.

Observed percentage agreement and the kappa statistic were used to measure agreement between AspLFD and the Platelia *Aspergillus* galactomannan assay at a cut-off index of ≥ 0.5 and ≥ 1.0. The strength of agreement was defined according to Altman’s scale as: < 0.20 (poor); 0.20 to 0.40 (fair); 0.40 to 0.60 (moderate); 0.60 to 0.80 (good); and 0.80 to 1.00 (very good agreement).

Any *P* value less than 0.05 was interpreted as statistically significant.

The Statistical Package for Social Sciences Software (SPSS version 25.0; IBM SPSS statistics Inc., Chicago, IL, USA) was used for statistical analysis. The local research ethics committees approved this study.

## Results

**Fig. 1:**
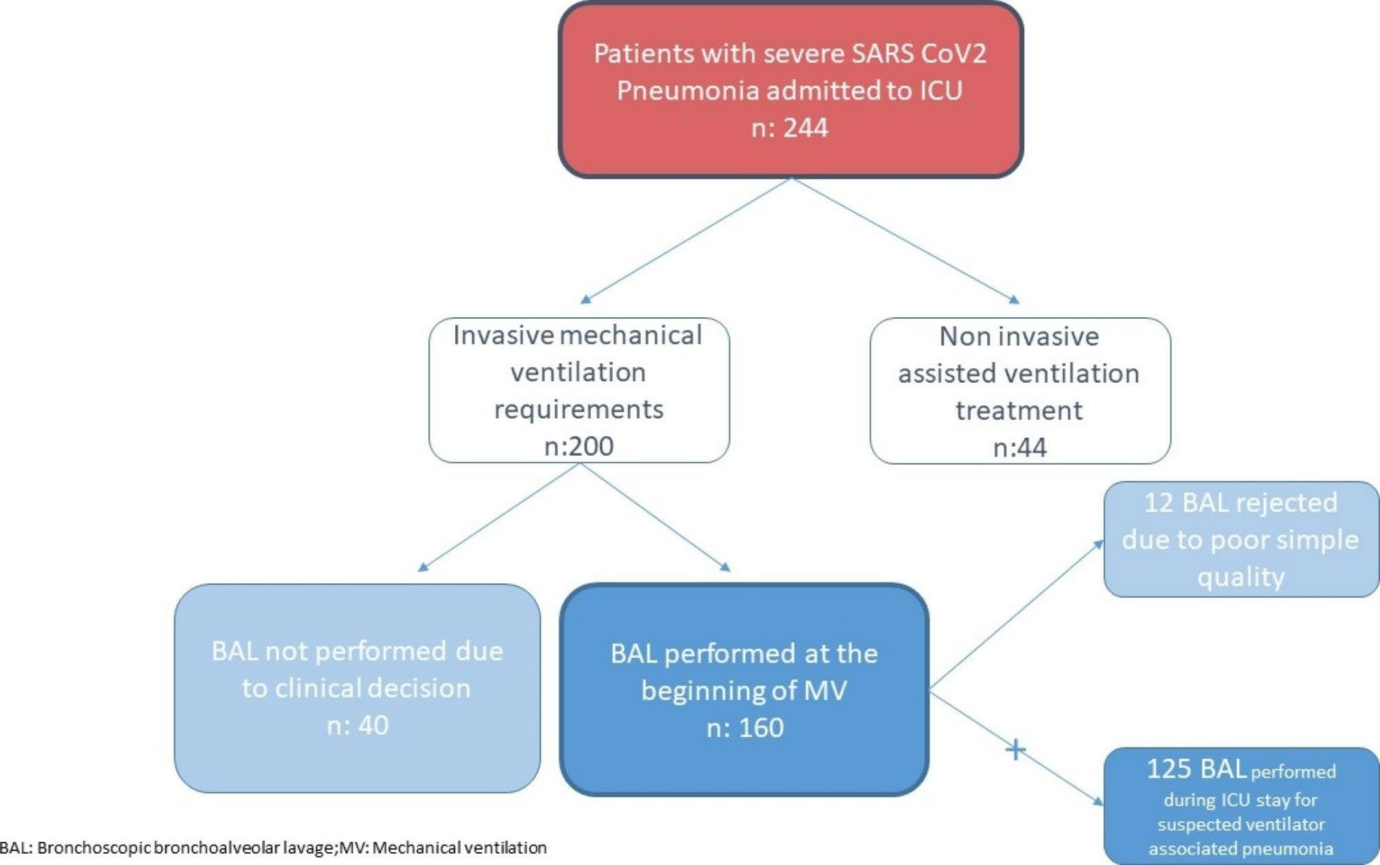
Flowchart of patients with severe SARS-CoV-2 pneumonia admitted to the ICU

A total of 244 patients with severe SARS-CoV-2 pneumonia were admitted to the ICU; 200 patients (82%) underwent mechanical ventilation, and diagnostic fiberoptic bronchoscopy was performed in 160 (80%) patients. During the study period, a total of 297 BAL fluid samples were collected; bronchoscopy was repeated in several patients during their ICU stay, indicated by clinical suspicion of ventilator-associated pneumonia. Finally, 285 BAL samples from 160 patients were selected for analysis (Fig. 1). We found 30 patients with CAPA (18.75%) and 130 cases without CAPA. Baseline characteristics are summarized in Table 1. Baseline characteristics of patients with and without CAPA were similar.

**Table 1:** Baseline characteristics of SARS-CoV-2 infected patients included in the study at ICU admission

Variable	n	Overall	No CAPA (*n = 130*)	CAPA	*P-value*
		(*n = 160*)		(*n = 30*)	
**Demographic variables**					
Age (years)	160	65 [57–71]	65 [57–71]	65 [58–70]	0.92
Male gender	160	115 (72%)	93 (71%)	22 (73%)	0.844
**Coexisting conditions**					
Hypertension	160	99 (62%)	79 (61%)	20 (67%)	0.549
Diabetes					
Obesity (BMI > 30)	160	51 (32%)	40 (31%)	11 (37%)	0.55
Heart disease	160	26 (16%)	17 (13%)	9 (30%)	0.05
Chronic kidney disease	160	13 (8%)	10 (8%)	3 (10%)	0.707
Liver disease	160	9 (6%)	6 (5%)	3 (10%)	0.371
COPD	160	6 (4%)	4 (3%)	2 (7%)	0.313
Asthma	160	7 (4%)	5 (4%)	2 (7%)	0.616
Chronic neurological disease	160	11 (7%)	11 (8%)	0 (0%)	0.221
Active malignancy	160	10 (6%)	8 (6%)	2 (6%)	1
Prior transplantation	160	7 (4%)	5 (4%)	2 (7%)	0.616
Immunosuppression	160	2 (1%)	1 (1%)	1 (3%)	0.341
**Laboratory analysis**					
*Blood counts*					
-Leukocytes [G/I]	157	11.2 [7.6–15]	11.2 [7.4–15.2]	11.8 [8.9–13.9]	0.585
-Neutrophils [G/I]	157	10.2 [6.9–14]	10.1 [6.5–12.9]	11.2 [8.0-14.1]	0.292
-Lymphocytes [G/I]	157	0.6 [0.5–0.9]	0.7 [0.5–0.9]	0.6 [0.4–1.1]	0.927
-Thrombocytes [G/I]	157	241 [180–313]	243 [181–319]	237 [172–310]	0.698
**Specific Medication**					
Glucocorticoids	139	121 (87%)	97 (86%)	24 (92%)	0.525
Remdesivir	139	11 (8%)	7 (6%)	4 (15%)	0.125
Lopinavir/ritonavir	139	27 (19%)	21 (19%)	6 (23%)	0.602
Tocilizumab	139	24 (17%)	22 (19%)	2 (8%)	0.248

Abbreviations: ICU intensive care unit; COPD chronic obstructive pulmonary disease; CAPA coronavirus disease-associated pulmonary aspergillosis

**Fig. 2:**
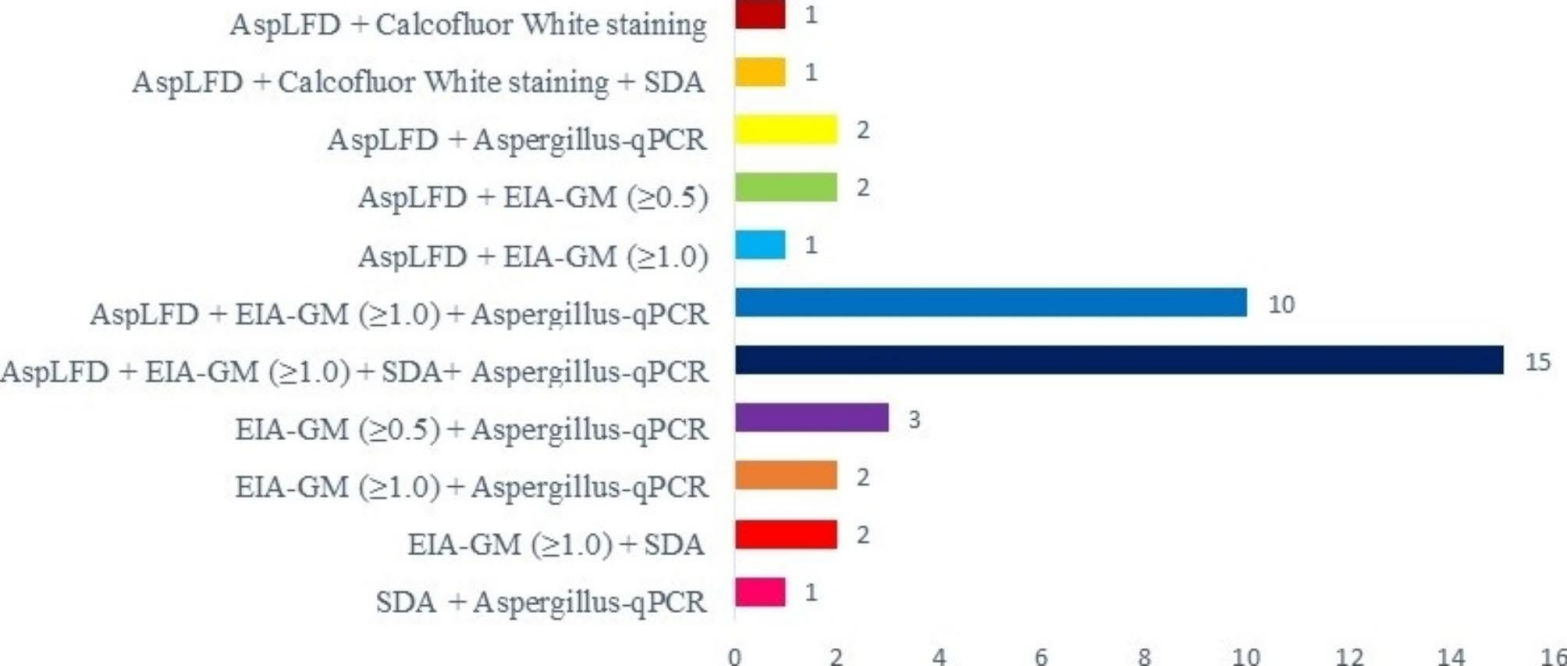
Combinations of the different diagnostic microbiological tests on positive BAL samples from patients with CAPA. Abbreviations: SDA Sabouraud dextrose agar;

A total of 40 BAL fluids from 30 patients with probable CAPA were positive. By AspLFD, 32/38 (84.2%) BAL samples tested positive. Culture on SAD with chloramphenicol was positive in 19/40 (47.5%) BAL samples, but only one with a positive calcofluor white stain. The GM-EIA (≥ 1) was positive in 30/38 (78.9%) BAL samples, and GM-EIA (≥ 0.5) in 35/38 (92.1%). Twenty-eight of 36 BAL samples (77.7%) showed positivity by both AspLFD and GM-EIA. All combinations of the positive diagnostic microbiological tests performed on BAL samples are shown in Fig. 2.

### AspLFD

Sensitivity and specificity were 84% and 99%, respectively (Table 2). There were 2 false positives (2/34) and 6 false negatives (6/236). The two false positives were negative by the Platelia *Aspergillus* galactomannan assay (≤ 0.5) and culture on SDA with chloramphenicol. The false negative results were all positive by *Aspergillus*-qPCR. Five of these were found to have a BAL GM value > 0.5, and the only one at the GM index of ≤ 0.5 was BAL culture-positive for *A. terreus* complex.

**Table 2:** Performance characteristics of the diagnostic tests used to diagnose CAPA

Test	TP	TN	FP	FN	S	E	PPV	NPV	LR(+)	LR(-)
**EIA-GM index ≥ 0.5**	35	205	11	3	92	95	76	99	18	0.08
**EIA-GM index ≥ 1.0**	30	213	3	8	79	99	91	96	79	0.21
**AspLFD**	32	230	2	6	84	99	94	97	84	0.16
**Cultured on SDA**	19	239	6	21	48	98	76	92	24	0.53
**Calcofluor white staining**	3	135	0	30	9	100	100	82	∞	0.91

Abbreviations: SDA sabouraud dextrose agar; TP true positive; TN true negative; FP false positive; FN false negative; S sensitivity; E specificity; PPV positive predictive value; NPV negative predictive value; LR(+) positive likelihood ratio; LR(-) negative likelihood ratio

### Calcofluor white staining

**Fig. 3:**
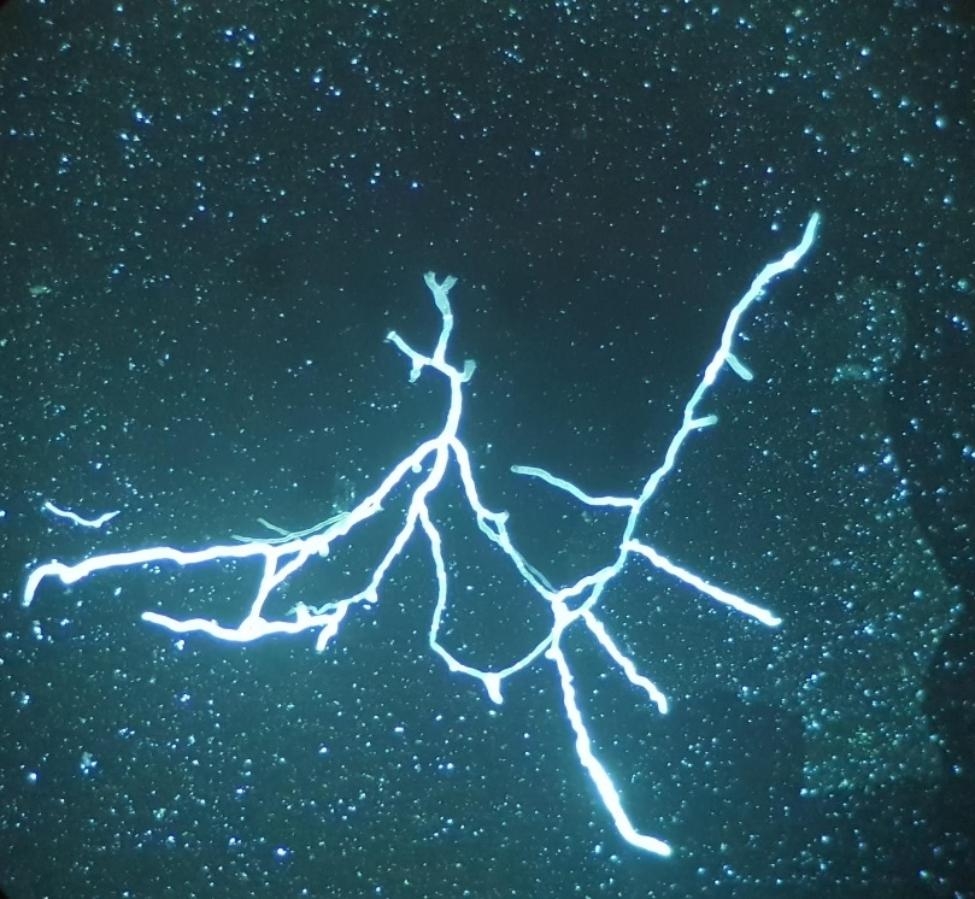
Calcofluor white staining suggestive of *Aspergillus* species in a critically ill patient with probable CAPA

Only three cases were positive. In all cases, AspLFD was positive. Two patients had *Aspergillus* on SAD culture (*A. terreus* complex and *A. flavus* complex). Sensitivity was 9% and specificity 100%. Figure 3 shows an image with positive calcofluor white stain.

### Culture on SDA with chloramphenicol

*Aspergillus* species isolated from fungal cultures in 13/30 patients (43.3%) with CAPA were: *Aspergillus terreus complex* in three patients (23%), *Aspergillus niger complex* in four (31%), *Aspergillus flavus complex* in four (31%) and *Aspergillus fumigatus complex* in two patients (15%). In 6 patients, different *Aspergillus* species were isolated (2 with *A.* fumigatus, 2 with *A. niger*, 2 with two or three different *Aspergillus* species) and were considered contaminations because all other tests were negative, including *Aspergillus*-qPCR. The median optical density index by Platelia *Aspergillus* galactomannan assay was 3.17 (2.95–3.88) from 19 determinations. Only one BAL sample with growth of *Aspergillus terreus* complex was negative for GM in both the AspLFD and the Platelia *Aspergillus* assay (GM = 0.49). This case was considered positive by *Aspergillus*-qPCR. The culture showed a sensitivity of 48% and a specificity of 98% (Table 2).

### Platelia ***Aspergillus*** galactomannan assay

At a cut-off of 0.5, BAL sensitivity and specificity were 92% and 95%, respectively (Table 2). Eleven false positive results (11/46) were found, 10 of which were culture-negative for *Aspergillus* species, and all were negative by *Aspergillus*-qPCR. Three results were false negative (3/208). In these patients, *Aspergillus*-qPCR was positive.

At a cut-off of 1.0, BAL sensitivity and specificity were 79% and 99%, respectively. Three false positives (3/33), with values between 1.0 and 1.3, were negative in all tests. Platelia GM showed 8 false negative cases (8/221), all with a positive *Aspergillus*-qPCR (Table 3).

**Table 3:** Performance characteristics of the Platelia *Aspergillus* galactomannan assay in BAL fluid

OD index cut-off	Parameter value (95% CI)			
	Sensitivity (%)	Specificity (%)	PPV (%)	NPV (%)
≥ 0,3	94.7 (82.2–99.3)	87.5 (82.3–91.6)	57.1 (44.0-69.5)	98.9 (96.3–99.9)
≥ 0.5	92.1 (78.6–98.3)	94.9 (91.1–97.4)	76.1 (61.2–87.4)	98.6 (95.8–99.7)
≥ 1.0	78.9 (62.7–90.4)	99.1 (96.7–99.9)	93.7 (79.2–99.2)	96.4 (93.0-98.4)
≥ 1.5	65.8 (48.6–80.4)	100 (98.3–100)	100 (90.7–100)	94.3 (90.5–96.9)
≥ 2.0	60.5 (43.4–75.9)	100 (98.3–100)	100 (85.2–100)	93.5 (89.5–96.3)
≥ 3.0	50 (33.4–66.6)	100 (98.3–100)	100 (82.3–100)	91.9 (87.7–95.1)
≥ 4.0	15.8 (6.0-31.2)	100 (98.3–100)	100 (54.1–100)	87.1 (82.3–91.0)

**Fig. 4:**
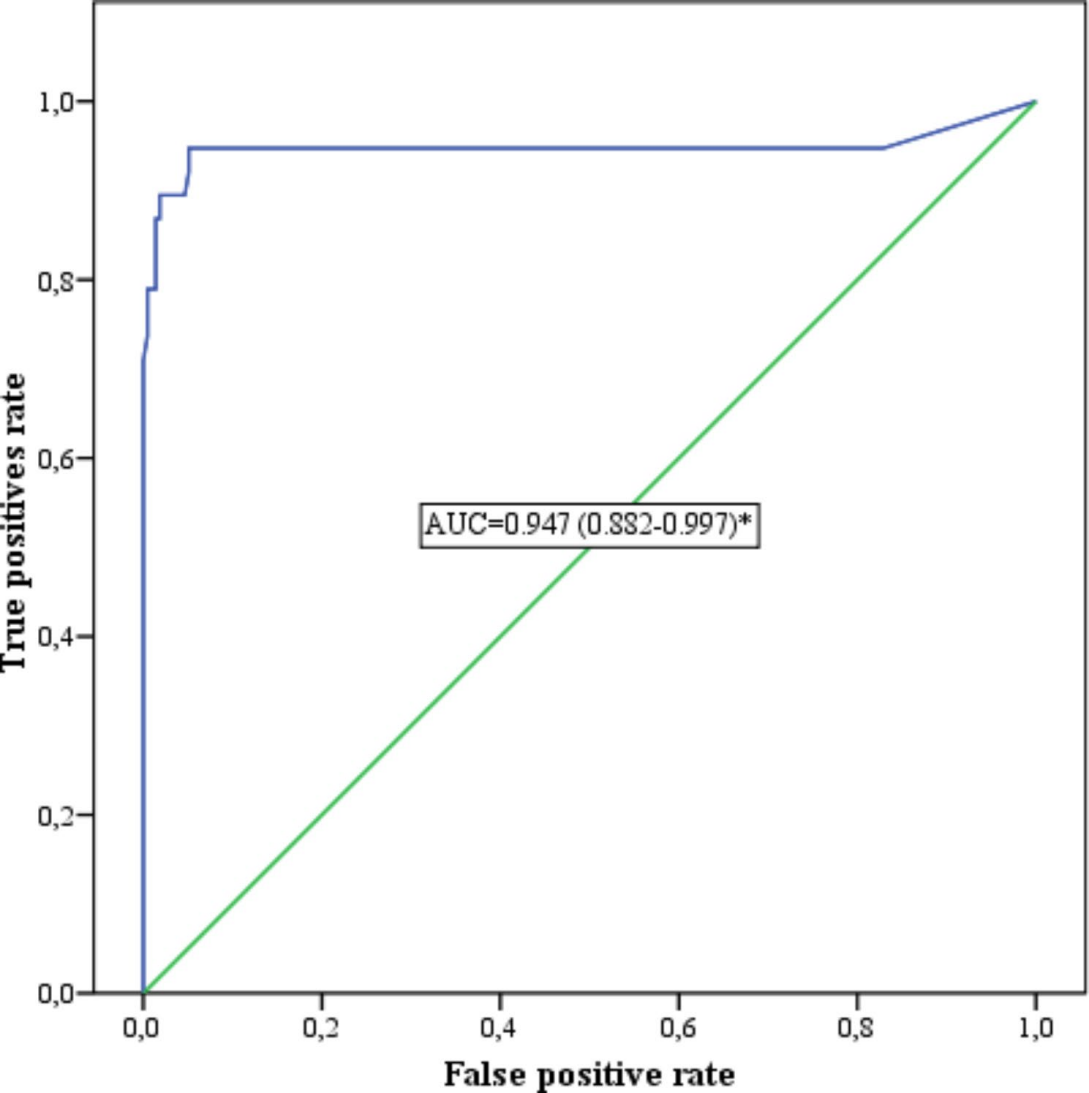
ROC analysis illustrating the optimal cut-off value for the Platelia *Aspergillus* galactomannan assay (AUC = 0.947 (0.882–0.997).* *p-value < 0.0001*

The optimal cut-off index for this assay in ROC curve analysis was 0.48, with 95% sensitivity and 95% specificity. The AUC of the ROC curve was 0.947 (95% CI: 0.882 to 0.997) (Fig. 4).

With an index ≥ 0.5, agreement between AspLFD and the Platelia *Aspergillus* galactomannan assay was good (kappa coefficient = 0.69). Positive percent agreement was 87% (28/32) and negative percent agreement was 92% (191/207).

At an optical density index ≥ 1.0, the comparative evaluation of AspLFD and the Platelia *Aspergillus* galactomannan test showed substantial agreement, demonstrating good concordance (kappa coefficient = 0.79). Positive percent agreement was 81% (26/32) and negative percent agreement was 98% (202/207).

## Discussion

Diagnosing CAPA in the ICU presents a real challenge, not only because of the non-specificity of the clinical and radiological criteria, which are present in many other causes of respiratory deterioration in severe SARS CoV2 pneumonia, but also because of the heterogeneity of respiratory samples indicated for the diagnosis of aspergillosis.

Performance of fiberoptic bronchoscopy should also be promoted in order to obtain good quality invasive samples. It is glaringly obvious that an active search for this complication should be made [13], supported by a microbiological diagnosis. This will not be obtained by a single microbiological test, but rather by applying all the methods available at each center [17]. We observed the presence of false negatives, which can be avoided or minimized by using several diagnostic methods as a panel. False positive Aspergillus galactomannan immunoassays coul be associated with intravenous human immunoglobulin administration, cross-reactivity with other fungi (e.g. *Cryptococcus* species, *Trichosporon* species, and *Fusarium* species), antibiotics (e.g. tazobactam) and blood products, nutritional support, and even food taken during the hospitalization. Criteria for clinical suspicion of pulmonary aspergillosis associated with SARS-CoV-2 are defined as fever not explained by any other cause and/or impaired respiratory function, as measured by paO2/FiO2. The radiological criteria include the presence of pulmonary infiltrate on a simple chest x-ray. Since these are common criteria for SARS-CoV-2 infection itself, it is essential to base it on a microbiological diagnosis.

The diagnostic criteria for CAPA are based on the 2020 ECMM/ISHAM consensus criteria. Detection of fungal elements in BAL indicating a mold, positive BAL culture, BAL GM index ≥ 1.0 or BAL LFA index ≥ 1.0, and a single positive *Aspergillus*-qPCR in BAL (< 36 cycles) are sufficient criteria to diagnose CAPA [16]. The use of a single diagnostic marker could overestimate the diagnosis of CAPA. In this study, we used a combination of two or more microbiological markers for diagnosis. The incidence of CAPA in this prospective cohort of 160 patients was 18.75%, which is similar to that found in several studies [18, 19]. By applying the 2020 ECMM/ISHAM consensus criteria however, the incidence in this study would have been 24.4% (n = 39). Nine patients with a CAPA diagnosis were ruled out by other microbiological markers. Three patients with a BAL GM index ≥ 1.0 were negative by AspLFD, SDA culture and *Aspergillus-*qPCR, and 6 patients with SDA culture of *Aspergillus* species returned negative results by AspLFD, the Platelia *Aspergillus* galactomannan assay and *Aspergillus-*qPCR.

In critically ill patients admitted to the ICU with SARS-CoV-2 infection, CAPA is a serious complication that increases mortality [20]. In these patients, rapid diagnosis is very important as it allows us to treat them with antifungal therapy in the first days after infection. Current diagnostic techniques, such as BAL culture and the GM-EIA are very slow and lead to a delay in diagnosis. AspLFD is a rapid test that has not been tested in this type of patient. There is documented experience in hematological patients [20, 21], but hardly any studies to validate it in the critically ill patient [22, 23], which is why it is not included in the ECMM/ISHAM consensus criteria. In this study, we analyzed the sensitivity and specificity of AspLFD in CAPA patients, which were 84% and 99%, respectively. Other lateral flow assays (LFA) such as the IMMY sōna AGM LFA showed lower sensitivity and specificity, 60% and 89%, respectively [24]. The use of BAL culture as a gold standard has several drawbacks, such as the low yield of fungal cultures in invasive aspergillosis [25] and culture media contaminated with *Aspergillus* spores present in the environment. Invasive samples are more reliable for the diagnosis of aspergillosis. As is well known, *Aspergillus* species are common colonizers of the respiratory tract, and their isolation in other samples such as tracheal aspirate culture could simply be colonization. In our study, only 19/34 (56%) patients had positive BAL cultures for *Aspergillus* species, although four *Aspergillus* samples were considered contaminated since they had negative *Aspergillus*-qPCR results and were negative by both GM-EIA and AspLFD. These results are lower than in another recent study, in which 22/33 (66.6%) patients were BAL culture-positive for *Aspergillus* species [24], although they did not show the results of the GM-EIA or GM-LFA in a positive BAL culture for *Aspergillus species* and so may be overestimated. In our study, the BAL culture yield was the third lowest of the mycological tests and only direct examination with calcofluor white staining had a lower performance. These results are similar to those reported in a cohort of patients with hematologic disease and invasive pulmonary aspergillosis, in which only 2/67 (3.6%) BAL fluid samples were positive in direct smears stained with calcofluor white [26]. The invasive diagnostic strategy in mechanically ventilated patients is not widespread in the ICU, due to lack of resources or trained personnel, and/or for professional safety reasons, such as fear of contagion in patients with SARS CoV-2 infection. We believe that this technique is safe and provides the clinician with valuable information that goes beyond the diagnosis of aspergillosis, bacterial coinfections and other pulmonary complications can also be explored as bronchoscopy allows a direct view of the interior of the airways. Since the availability of different methods of microbiological diagnosis varies between centers and advances in the diagnostic arsenal remain insufficient, making it difficult to detect invasive aspergillosis in the early stages of severe SARS-CoV-2 pneumonia, an active search is recommended, combining the procedures available.

This study has some limitations. This was a real clinical practice study conducted in a single center, and therefore requires further studies with the participation of more ICUs to confirm the findings. As this was a clinical study, the determination of galactomannan in serum was not taken into account, given that the patients were not neutropenic. It would be interesting to know what the outcome would be in a patient population with severe SARS-CoV-2 pneumonia, as this has not been studied previously. The main strength of this study in patients with severe SARS-CoV-2 pneumonia was the invasive diagnostic strategy used, despite the fact that, at the beginning of the pandemic, fibrobronchoscopy was not recommended by different societies for fear of generating aerosols. Since the beginning of the pandemic, BAL was performed after intubation in most patients. Another novelty is that we tested a diagnostic method like lateral flow in critically ill patients with suspected CAPA. This may be advantageous for early treatment of a disease associated with high mortality, in which the microbiological diagnosis often takes time to be confirmed.

## Conclusion

Using a diagnostic strategy based on bronchoscopy and BAL, we documented a high incidence of pulmonary aspergillosis in severe SARS-CoV-2 pneumonia patients. This is the first study to analyze AspLFD as a mycological marker for the diagnosis of CAPA. The sensitivity was not very high, but its positive predictive value was 94%, which could be helpful for rapid diagnosis of this disease. A delay in the diagnosis of CAPA is associated with a poor prognosis and increased mortality. In addition, the combination of several mycological markers improved the diagnosis of CAPA by reducing the false positives associated with diagnostic tests.

## Data Availability

The datasets used and/or analyzed during the current study are available from the corresponding author on reasonable request.
