# Correction: Microbiological diagnosis of pulmonary invasive aspergillosis in critically ill patients with severe SARS-CoV-2 pneumonia: a bronchoalveolar study

**DOI:** 10.1186/s12941-023-00648-1

**Published:** 2023-11-09

**Authors:** Ángel Estella, Ignacio Martín-Loeches, María Recuerda Núñez, Clara González García, Liliana Marcela Pesaresi, Alvaro Antón Escors, Maria Dolores López Prieto, Juan Manuel Sánchez Calvo

**Affiliations:** 1 Intensive Care Unit University Hospital of Jerez, University of Cádiz. INIBiCA, Jerez de la Frontera, Spain; 2 Department of Intensive Care Medicine, Multidisciplinary Intensive Care Research Organization (MICRO), St James’ Hospital, Dublin, Ireland; 3 Intensive Care Unit University Hospital of Jerez, INIBiCA, Jerez de la Frontera, Spain; 4 Medical School University of Cádiz, Cadiz, Spain; 5 Infectious diseases and Microbiology, Unit Hospital Universitario de Jerez, INIBiCA, Jerez de la Frontera, Spain

Correction to: Annals of Clinical Microbiology and Antimicrobials (2023) **22**:90

10.1186/s12941-023-00626-7

Following publication of the original article [1], the author name “Ignacio Martín-Loeches” was incorrectly written as “Ignacio Martin Loeches”. This has now been corrected with this erratum.

The original article has been corrected.

